# Learning Pitch with STDP: A Computational Model of Place and Temporal Pitch Perception Using Spiking Neural Networks

**DOI:** 10.1371/journal.pcbi.1004860

**Published:** 2016-04-06

**Authors:** Nafise Erfanian Saeedi, Peter J. Blamey, Anthony N. Burkitt, David B. Grayden

**Affiliations:** 1 NeuroEngineering Laboratory, Department of Electrical and Electronic Engineering, University of Melbourne, Melbourne, Victoria, Australia; 2 The Bionics Institute, East Melbourne, Victoria, Australia; 3 Department of Medical Bionics, University of Melbourne, Melbourne, Victoria, Australia; 4 Centre for Neural Engineering, University of Melbourne, Melbourne, Victoria, Australia; Radboud Universiteit Nijmegen, NETHERLANDS

## Abstract

Pitch perception is important for understanding speech prosody, music perception, recognizing tones in tonal languages, and perceiving speech in noisy environments. The two principal pitch perception theories consider the place of maximum neural excitation along the auditory nerve and the temporal pattern of the auditory neurons’ action potentials (spikes) as pitch cues. This paper describes a biophysical mechanism by which fine-structure temporal information can be extracted from the spikes generated at the auditory periphery. Deriving meaningful pitch-related information from spike times requires neural structures specialized in capturing synchronous or correlated activity from amongst neural events. The emergence of such pitch-processing neural mechanisms is described through a computational model of auditory processing. Simulation results show that a correlation-based, unsupervised, spike-based form of Hebbian learning can explain the development of neural structures required for recognizing the pitch of simple and complex tones, with or without the fundamental frequency. The temporal code is robust to variations in the spectral shape of the signal and thus can explain the phenomenon of pitch constancy.

## Author Summary

Pitch is the perceptual correlate of sound frequency. Our auditory system has a sophisticated mechanism to process and perceive the neural information corresponding to pitch. This mechanism employs both the place and the temporal pattern of pitch-evoked neural events. Based on the known functions of the auditory system, we develop a computational model of pitch perception using a network of neurons with modifiable connections. We demonstrate that a well-known neural learning rule that is based on the timing of the neural events can identify and strengthen the neuronal connections that are most effective for the extraction of pitch. By providing an insight into how the auditory system interprets pitch information, the results of our study can be used to develop improved sound processing strategies for cochlear implants. In cochlear implant hearing, auditory percept is generated by stimulating the auditory neurons with controlled electrical impulses, enhancing which with the help of the model would lead to a better representation of pitch and would subsequently improve music perception and speech understanding in noisy environments in cochlear implant users.

## Introduction

The existence of a pitch processing center or a group of specialized “pitch neurons” in the mammalian auditory system has been debated in recent years. For example, through single unit recordings, Bendor and Wang [1] found a potential pitch center in the anterolateral border of primary auditory cortex in marmoset monkeys. These pitch neurons were characterized by sustained spiking in response to their preferred pitch, evoked by a pure tone or a harmonic complex. Human brain analogues of monkey’s lateral primary auditory cortex, postulated by Bendor and Wang [1] to be the pitch center, has also been found to perform pitch-related processing. For example, through positron emission tomography (PET), Zatorre and Belin [2] found that areas in the lateral Heschl’s gyrus responded to the pitch of pure tones. Using functional magnetic resonance imaging (fMRI), Patterson et al. [3] found the same cortical area to be consistently activated by periodic stimuli with a defined pitch. Penagos et al. [4] also confirmed the sensitivity of the Heschl’s gyrus area to the pitch of harmonic complexes through fMRI investigations.

Possible locations for pitch sensitive neural units along the auditory pathway have been postulated in a number of modelling studies. For example, the coincidence detector neurons of the model of Shamma and Klein [5] required strong phase-locked inputs; therefore, Shamma and Klein proposed the inferior colliculus as a possible pitch processing site. Inferior colliculus neurons receive inputs from the cochlear nucleus neurons that, due to having onset type cells [6], generate spectrally and temporally sharp responses suitable for coincidence detector units.

The functional role of the cochlear nucleus in varying the timing of spikes has been observed in earlier studies [7]. Spike time variation in the auditory nerve is partially caused by the cochlear travelling wave and results in the spiking of neurons with high characteristic frequency (CF) several milliseconds prior to low-CF neurons in response to a stimulus. Through experimental studies, Oertel et al. [8] showed that it was particularly octopus cells in the cochlear nucleus that had the ability to detect spiking coincidences among a population of innervating auditory nerve fibers. The octopus cells were found to compensate for the different arrival times of the auditory nerve spikes. The ability of octopus cells to extract precise temporal information from the auditory nerve was related to their special anatomical structure and biophysical characteristics [9]. The compensating role of octopus cells was further investigated in a modelling study by Spencer et al. [10]. They showed that different arrival times of the auditory nerve spikes were compensated by proportional dendritic delays in the octopus cells, thus enabling the detection of the spike coincidences to be carried out more effectively in later stages.

Given the uncertainties that still exist about the physiology of pitch centers in the auditory system, the focus of this paper is on modelling the known *functions* of the possible pitch neurons rather than replicating the anatomical stages (and their interactions) involved in extracting the pitch information. According to existing literature, pitch sensitive neurons have a preferred pitch [11], exhibit sustained spiking activity [12,13], respond to pitch as a unified entity (regardless of the spectral shape of the stimuli) [1], and are located in the subcortical part of the auditory pathway [5,7].

In the model developed in this paper, it was assumed that the spectral (place) and temporal pitch information were processed by different populations of neurons. These neuronal populations, despite being connected to each other, used different mechanisms to extract their component of pitch information. One reason for considering such a neuronal architecture was the functioning differences between the two hemispheres of the brain in processing the pitch of stimulus. For example, Zatorre and Belin [2] found that the right hemisphere exhibited a stronger response to pitch-related spectral variations, while the left hemisphere showed higher degrees of activation in response to temporal variations of the stimulus. Based on these observations, Zatorre and Belin [2] suggested that the auditory system had two parallel processing sub-systems that provided different spectral and temporal resolutions required for perceiving a wide range of stimuli, such as speech and music. Poeppel [14] proposed that the hemispheric functional differences were a result of different timescale integration windows applied by each hemisphere (i.e., shorter for the left and longer for the right hemisphere) when processing auditory information. In a lesion study, Johnsrude et al. [15] identified the right Heschl’s gyrus as responsible for making judgments on the direction of pitch changes (i.e., pitch ranking) because patients whose right temporal lobes were partially resected showed higher pitch-difference thresholds compared to the control group. They also found that, unlike pitch ranking, a pitch discrimination task (detecting a pitch difference regardless of direction) could be performed by either hemisphere.

**Fig 1:**
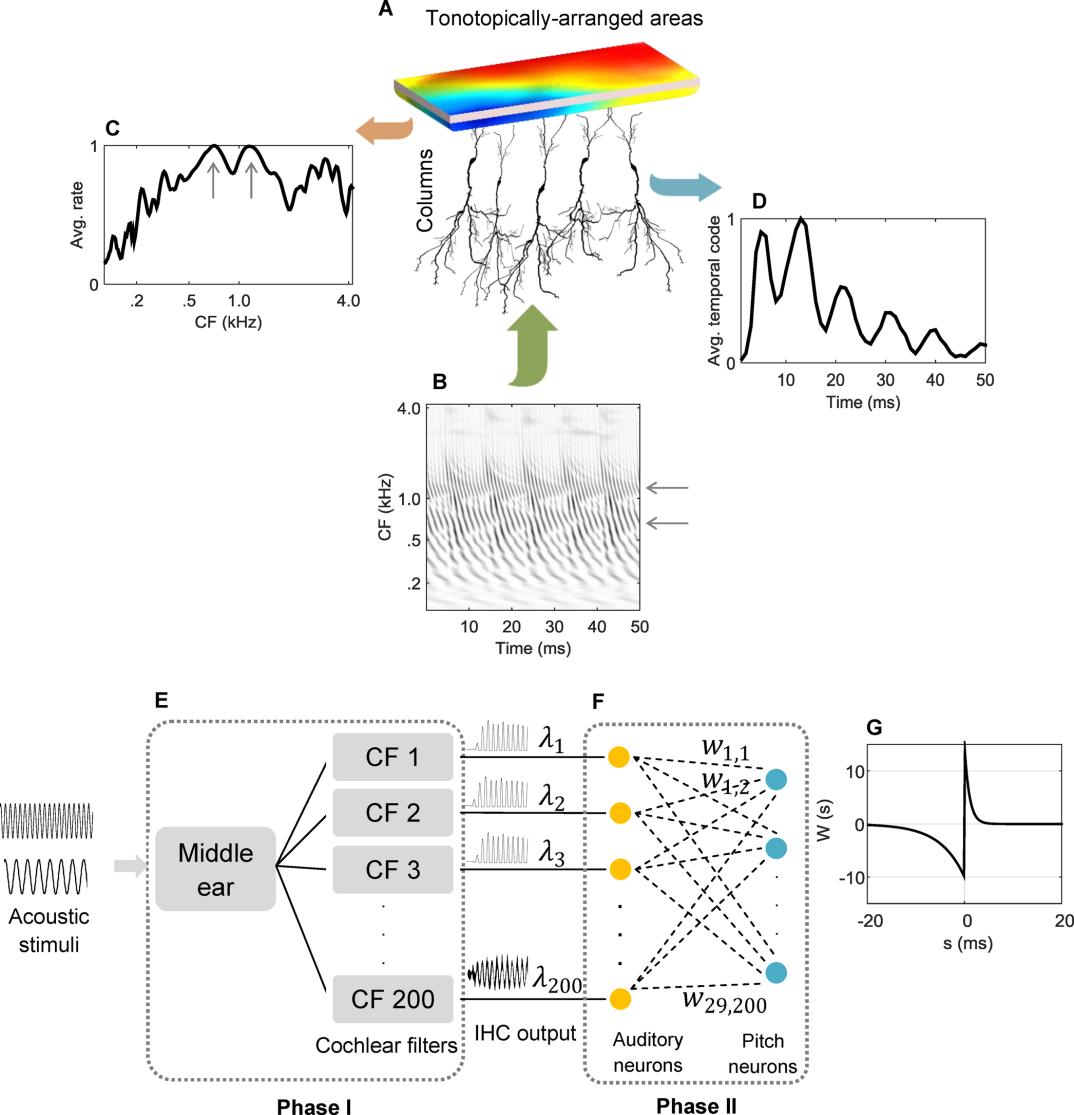
Cortical structure consisting of tonotopically-arranged areas of columns and their expected processed outputs. (A) Tonotopically-arranged areas of columns. Colors of the exemplar tonotopicity represent tuning to different frequencies. (B) A spatio-temporal map for a periodic stimulus is generated by the auditory nerve and provided to the next processing level. The stimulus is a synthesized /ɑ/ vowel, with F0 = 110 Hz and the first three formants at 710 Hz, 1150 Hz, and 2700 Hz indicated with grey arrows. Dark areas show stronger activities. (C) The average neural activity as a measure of the signal power originates from the tonotopical arrangement. Dominant spectral peaks (grey arrows) correspond to the first two vowel formants. (D) Expected extracted temporal code from the columns that would serve as a measure of phase or synchrony between the neurons. The stimulus period is manifested through the inter-peak time intervals and their relative amplitudes. Both spectral and temporal codes are normalized to maximum for better visualization. (E-F) Computational analogue of the cortical structure shown in (A). Phase I and Phase II are responsible for extracting the place and temporal pitch codes, respectively. A model of the auditory periphery constitutes Phase I. The outputs of Phase I are converted into spike times and input to Phase II. The input/output synapses of the spiking neural network in the second phase are modified by a neural learning algorithm in order to generate precisely-timed spike trains in the output, from which a meaningful code for pitch can be extracted. (G) The shape of the STDP learning window used in the learning rule described in the Methods section. The notation shown in the figure is used in the learning equations presented in the Methods section.

Another observation that inspired the use of a separate population of neurons for temporal pitch processing in the auditory system is the special organization of brain tissue [16] as illustrated in Fig 1A. Inputs to the auditory cortex can be presented in terms of spatio-temporal maps that describe the activity of spatially different neurons over time [17]. Fig 1B shows an example of a spatio-temporal pattern for a synthesized vowel /ɑ/, with F0 of 110 Hz and the first three formants located at 710 Hz, 1150 Hz, and 2700 Hz, using a model of auditory periphery developed by Zilany et al. [18]. Observations have shown that tonotopicity (viz., neurons responding to a frequency based on their location, leading to a place-frequency map) exists in the areas of higher auditory processing centers like the auditory cortex [19]. Tonotopicity (indicated by the color map in Fig 1A) thus accounts for the extraction of power-based or place features from the signal. Spectral features including the first two formants are strongly represented in the spatio-temporal patterns. The first two formants are indicated with grey arrows in Fig 1B. The role of the tonotopically-arranged areas could be interpreted as averaging the spatio-temporal patterns over time, resulting in a profile of activity rates across the auditory nerve. Fig 1C shows the corresponding rate profile (normalized to maximum) that is considered as the place code of pitch. The first two formants (indicated with grey arrows) have strong representation in the place code shown in Fig 1C. The cortex also has columnar divisions with connections to the tonotopically-arranged areas. According to this area-column synergy, measuring the activity across columns would provide a temporal code for pitch. Fig 1D represents a possible temporal code, extraction of which is the topic of this paper. Of note is the spacing between the peaks of the temporal code in Fig 1D that corresponds to the period of stimulus (i.e., ~9 ms), which is the F0 of the vowel.

Unlike the place code, simple averaging would not capture the temporal code because of the temporal variations (e.g., jitter) that naturally exists in the neural code generated by the auditory nerve. Therefore, capturing the fine-time structures, such as spike coincidences, from the neural code required an intermediate processing stage that adjusted the spike timings before any sort of averaging occurred. This intermediate processing stage would possibly replicate the functional role of the cochlear nucleus [7]. Enabling a model of cochlear nucleus to perform this function required specific neural connectivity that could arise through neural plasticity.

The computational analogue of the cortical structure shown in Fig 1A is depicted in Fig 1E and 1F. The two phases were defined by the set of modelling components that together extracted the place (Phase I) or temporal (Phase II) cues for pitch perception. Phase I performed temporal averaging for auditory neurons. Phase II provided a biologically-inspired computational substrate for producing precisely-timed spikes that would lead to an efficient temporal pitch code. A spiking neural network with plastic input/output synapses constituted the second phase. It is apparent that Fig 1C and 1D represent the outputs of Phase I and Phase II of the analogue shown in Fig 1E and 1F, respectively.

## Methods

This section describes the data used in the simulations and the process of place and temporal pitch information extraction. Extraction of the place code is described jointly with the model of auditory periphery in the Auditory periphery (Phase I) section. Extracting the temporal code requires a neural setup that is properly adjusted to fit the pitch perception task. The neural components and associated learning equations are presented in the Neural setup and Synaptic adjustments sections, respectively.

### Data

Sound stimuli for this study were synthesized and real-world sounds that a typical listener might experience. Synthesized sounds are advantageous because they can be generated easily and precisely. However, for the sake of generality, real-world recordings from various musical instruments were also included in the simulations. Types of stimuli and their descriptions are given in Table 1. All stimuli were 0.5 s long, had a loudness of 60 dB SPL, and were sampled at 16,000 sample/s.

**Table 1:** Types of sound stimuli and associated parameters used in this study.

	Type	# Samples	Parameters	Notation
**1**	Pure Tones	29	Sinusoids of 29 frequencies, spaced one semitone apart and covering the [98 Hz, 493 Hz] range.	—
**2**	Vowels	58	Sustained vowels /ɑ/ and /i/, synthesized using the cascade branch of the KLATT speech synthesizer [20]. The vowels’ first three formant frequencies (in Hz) and their associated bandwidths (shown in brackets, also in Hz) were 710 [40], 1150 [43], and 2700 [105] for /ɑ/ and 230 [68], 2000 [63], and 3000 [129] for /i/.	/ɑ/, /i/
**3**	Telephone Vowels	58	The synthesized vowels were filtered through a high-pass filter with a cut-off frequency of 300 Hz, simulating the effect of telephone transmission line.	/ɑ/_T_, /i/_T_
**5**	Musical Instruments	79	Includes 29 piano, 17 violin, 13 flute, and 20 cello recorded sounds with pitch labels matching those of the pure tones. All sound files were retrieved from the Electronic Music Studios of the University of Iowa (http://theremin.music.uiowa.edu/index.html). Only one recorded audio channel was used. The sampling rate was changed to 16,000 sample/s.	—

Pure tones were desirable stimuli because they have simple spectral shapes and evoke salient pitches. Speech is possibly the most common sound stimulus that one might experience; therefore, voiced speech tokens (sung vowels /ɑ/ and /i/) were well-suited to the purpose of this study. Synthetically generated variations of the vowel stimuli (/ɑ/_T_ and /i/_T_) were also included in this study. This enabled the investigation of how the auditory system encoded pitch in real-life listening conditions such as telephone conversation, wherein the low-frequency contents of speech, including the F0 for most speakers, would be eliminated. The telephone line was simulated by high-pass filtering the original vowels using a high-pass FIR (finite impulse response) filter with a sharp cut-off frequency of 300 Hz. The filter was designed using MATLAB Filter Design & Analysis Tool with Fstop = 300 Hz, Fpass = 350 Hz, Astop = 80 dB, and Apass = 1dB. Filter order was 110 (minimum) and sampling rate was 16,000 sample/s.

Musical instruments provide a variety of spectral shapes and were included in the simulations to investigate the behavior of the model in response to spectrally-different sounds. The instruments were selected based on availability in the database, spectral shape variety, and the range of pitches that each instrument could generate.

### Extraction of pitch information

The purpose of this study was to investigate how place and temporal pitch cues were extracted from the spatio-temporal maps generated by the auditory nerve. The former was assumed to be a profile of rates (temporal averages) associated with different auditory neurons. Extraction of the place cues is described jointly with the generation of the spatio-temporal maps in the Auditory periphery (Phase I) section. The Temporal code of pitch (Phase II) section explains the neural structure configuration and the associated learning process leading to pitch-related temporal information.

#### Auditory periphery (Phase I)

The auditory periphery was modelled by a middle ear filter, followed by a 200-channel cochlear filter bank. Each filter simulated a single cochlear position and its output was interpreted as the activity of an inner hair cell (IHC) with a characteristic frequency (CF) equal to the center frequency of the filter. The structure of the middle ear and cochlear filters are described by Zilany and Bruce [21]. In the simulations in this study, the updated implementation of the cochlear filters, available online at http://goo.gl/MCTzjT, was used. CFs were determined based on the cochlear positions that the filters represented using the Greenwood function [22],

$$CF\left(d\right)=165.4\times \left(10^{2.1\times \frac{d}{34}}-1\right),$$ (1)

where *d* is the position of the cochlear filter (measured from the apex of the cochlea in mm) and was incremented in 0.1 mm steps. Cochlear positions in the range 3–22.9 mm were modelled. This led to cochlear filters with center frequencies from 88 Hz up to nearly 4 kHz.

**Fig 2:**
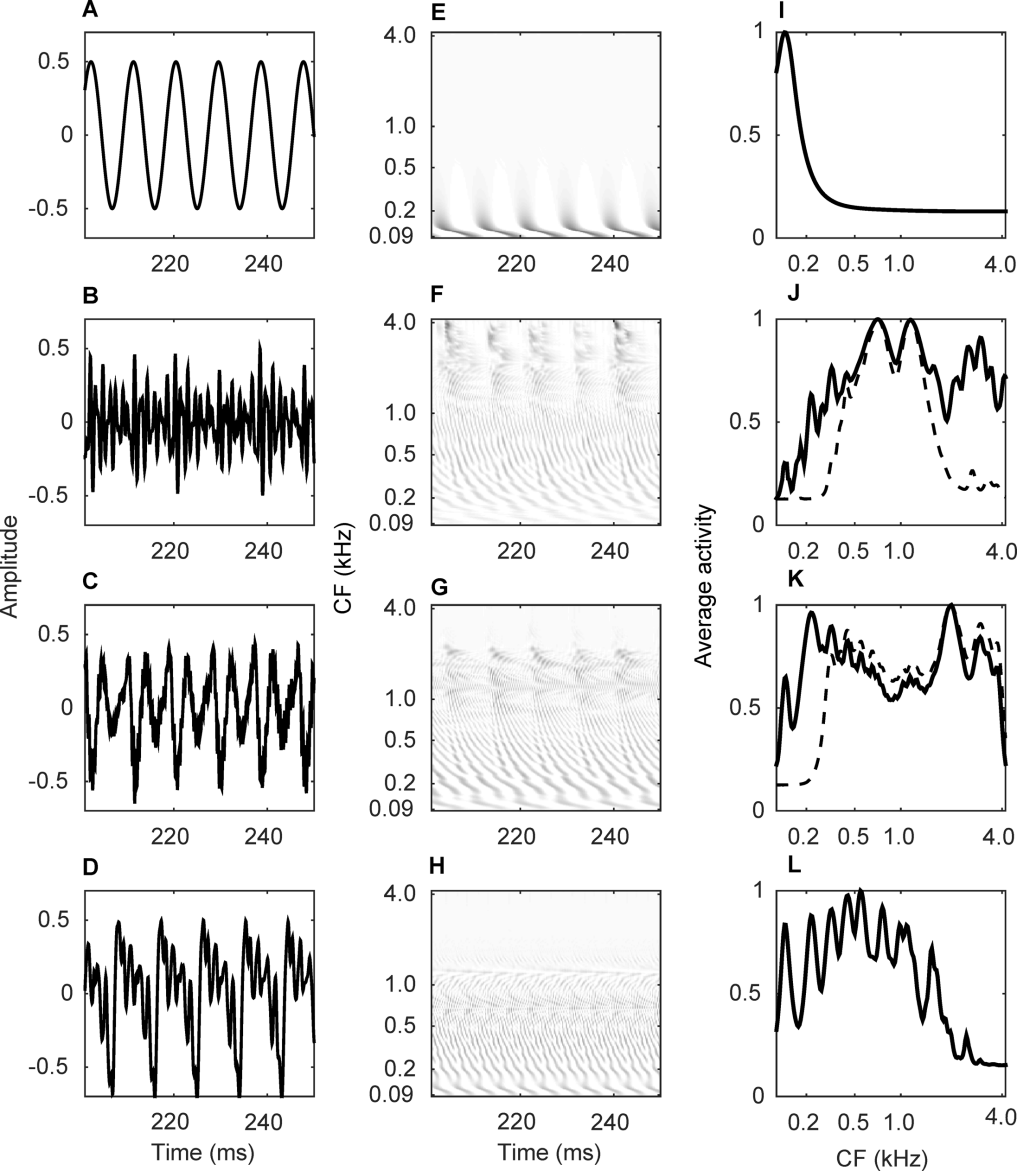
Acoustical waveforms and spatio-temporal maps for four types of stimuli eliciting a pitch of 110 Hz. (A) Temporal representation of a pure tone of 110 Hz. (B) Temporal representation of an /ɑ/ stimulus with F0 = 110 Hz. (C) Temporal representation of an /i/ stimulus with F0 = 110 Hz. (D) Temporal representation of a piano key with an absolute frequency of 110 Hz. (E) Spatio-temporal map for a pure tone of 110 Hz. (F) Spatio-temporal map for an /ɑ/ stimulus with F0 = 110 Hz. (G) Spatio-temporal map for an /i/ stimulus with F0 = 110 Hz. (H) Spatio-temporal map for a piano key with an absolute frequency of 110 Hz. Amplitude scale is arbitrary but consistent in (A-D). The 200 auditory neurons are sorted based on their CFs on the ordinate in (E-H). Dark areas show stronger activity in (E-H). (I) Averaged spatio-temporal map (extracted place code of pitch) for a pure tone of 110 Hz. (J) Averaged spatio-temporal map for an /ɑ/ stimulus with F0 = 110 Hz (solid line) and its high-pass filtered version (dashed). (K) Averaged spatio-temporal map for an /i/ stimulus with F0 = 110 Hz (solid) and its high-pass filtered version (dashed). (L) Averaged spatio-temporal map for a piano key with an absolute frequency of 110 Hz. Averaging interval is 100 ms long in (I-L) and resulting activities have been normalized to maximum for better visualization. The 200 auditory neurons are sorted based on their CFs on the abscissa in (I-L).

Spatio-temporal maps were generated by stacking the activity of the 200 tonotopically-ordered IHCs at different time steps. Fig 2 shows the temporal representation of the acoustical waveform (A-D) and simulated spatio-temporal maps (E-H) for pure tone, /ɑ/ stimuli, /i/ stimuli, and piano notes, respectively, all eliciting the same pitch of 110 Hz. Dark areas in the spatio-temporal maps show stronger rate of activity across cochlear positions, represented by their CF on the ordinate. The periodic behavior of the waveform is reflected in the spatio-temporal maps for the four stimuli.

For the pure tone, activities show a simple pattern repeating approximately every 10 ms (corresponding to the period of a 110 Hz tone) and are concentrated only at low-CF cochlear regions (towards the apex of the cochlea). For the two vowels, on the other hand, activity patterns are spread across a wider cochlear range and have a more complex periodic structure due to formants. The piano key sound activated lower-CF cochlear regions and the patterns were more oscillatory compared to the vowels due to the percussive nature of this instrument.

The place code of pitch refers to the rate of activity across different cochlear locations. To extract the rate of activity, the output of each cochlear filter was averaged over a 100 ms interval. This led to a vector of 200 rates for each sound stimulus. Extracted place code for the four stimuli presented in Fig 2A–2D are shown in Fig 2I–2L. The latter presents average activities or rate profiles as a function of cochlear position. The rate profiles derived from the pure tone (Fig 2I) has a single peak at a cochlear position with a CF similar to its frequency. The vowels (Fig 2J and 2K-solid lines) show a weak representation of the pitch-related low-frequency peak, plus formant-related peaks in the middle-frequency range. Consistent with the vowels’ characteristics, the formant-related peaks occur at approximately 700 Hz and 1100 Hz for /ɑ/ (Fig 2B) and at 230 Hz and 2000 Hz for /i/ (Fig 2C). The place codes associated with the telephone simulated vowels are shown with dashed lines in Fig 2J and 2K. It is observed that removing the low-frequency content of the signal suppresses the place code at these regions. For /ɑ/, this also affects the frequency region between the second and third formants. The place code of pitch is, therefore, highly dependent on the spectral shape of the stimulus. For the piano sound (Fig 2L), the extracted place code shows stronger dips and peaks compared to the vowels, indicating a clear-cut harmonic structure for this type of sound.

#### Temporal code of pitch (Phase II)

As shown in the Auditory periphery (Phase I) section, the auditory nerve contained information on pitch combined with other sound attributes such as spectral shape or timbre. In addition to pitch and timbre, loudness and sound source location also have neural representations [23,24]. The purpose of Phase II was to develop a biologically-plausible neural substrate that would enable capturing the pitch-related temporal information from the activity of the auditory neurons. The ultimate goal was to generate phase-locked responses at the output of Phase II so that averaging over pitch neurons would not lead to loss of temporal pitch information. Temporal adjustment of the post-synaptic spikes required employing a spiking interface and modifying the synaptic connections of the spiking neural network through a plasticity rule that was capable of capturing the correlated activity among pre-synaptic neurons. These two requirements are described in the following two sections.

#### Neural setup

Each auditory neuron was simulated by an inhomogeneous Poisson process with an intensity, ***λ***_***j***_, **1 ≤ *j* ≤ 200**, equal to the amplitude of IHC activity innervated by the auditory neuron. The IHC output was, therefore, interpreted as the time-dependent instantaneous rate of spike arrival at each synapse, indicated by ***w***_***ij***_, **1 ≤ *j* ≤ 200**. The refractory effect was included in the synapse model [21,25] and so was not included in the spike generation process.

The spike train generated by each auditory neuron was represented by

$$S_{j}\left(t\right)=\;\sum_{t_{j}^{\left(f\right)}}\delta (t-t_{j}^{\left(f\right)}),$$ (2)

where $t_{j}^{\left(f\right)}$ denotes individual spiking times for neuron *j* and *δ* is the Dirac delta function.

In the output layer of Phase II, 29 leaky integrate-and-fire (LIF) neurons received the spike trains generated by tonotopically-ordered auditory neurons through plastic synaptic connections. The dynamics of each LIF neuron was described by its membrane potential, *V*_*i*_(*t*), and was expressed in terms of all the synaptic currents that the neuron received [26],

$$\frac{dV_{i}\left(t\right)}{dt}=\;\frac{1}{\tau_{m}}\left(V_{p}-V_{i}\left(t\right)+\sum_{j}\left\{w_{ij}\left(t\right)\;\left[V_{rev_{j}}-V_{i}\left(t\right)\right]\;\sum_{f}\epsilon \left(t-t_{j}^{\left(f\right)}-\Delta_{j}\right)\right\}\right),$$ (3)

where *τ*_*m*_ is the membrane time-constant, *V*_*p*_ is the resting membrane potential, and $V_{rev_{j}}$ is the synaptic reversal potential for neuron *j*. The term $\frac{V_{p}-V_{i}\left(t\right)}{\tau_{m}}$ constitutes the leak current. The remaining terms on the right-hand side of (3) describe the input synaptic currents originating from the auditory neurons. *ϵ*(*t*) characterizes the shape of the post-synaptic conductance in the conductance-based LIF model considered in this study. Δ_*j*_ is the axonal delay for neuron *j*. The post-synaptic spikes were generated when the membrane potential crossed the threshold, *V*_*th*_, following which the membrane threshold was set to *V*_*r*_.

The weights specified the contribution of each auditory neuron in changing the membrane potential and evolved as learning (see the Synaptic adjustments section) proceeded. Inputs to the LIF neurons were all assumed to be excitatory and modulated by a double-exponential excitatory post-synaptic conductance (EPSC) kernel of the following form

$$\epsilon \left(t\right)=\;\frac{1}{\tau_{B}-\tau_{A}}\left(e^{-\frac{t}{\tau_{B}}}-e^{-\frac{t}{\tau_{A}}}\right)\;H\left(t\right),$$ (4)

where *τ*_*A*_ and *τ*_*B*_ are the EPSC rise time and decay time, respectively. *H*(*t*) is a Heaviside function, with *H*(*t*) = 1 for *t* ≥ 0 and *H*(*t*) = 0 otherwise.

#### Synaptic adjustments

Correlation-based plasticity rules have been widely used to describe the underlying processes that contributed to neural circuit development and memory storage (e.g., [27]). Spike-timing-dependent plasticity (STDP) is a well-known unsupervised correlation-based plasticity rule inspired by electrophysiological observations [28,29]. General STDP and its variations have been widely used in neural modelling studies. For example, Gerstner et al. [30] used STDP to explain the temporal precision of the spike times required for detecting the interaural time differences (~5 μs) in the nucleus laminaris of the auditory system in barn owls. They showed that STDP could successfully adjust the timing of the action potentials in an unsupervised fashion by identifying and strengthening the incoming synapses that had a particular delay, leading to a high temporal precision.

Hebbian learning requires both pre- and post-synaptic neurons to be active simultaneously for a synaptic change to take place [31]. STDP provides a reformulation of the simplified rate-based Hebbian rule to account for temporal correlation aspects of learning, such as the pre- and post-synaptic spike-timing coherency that is required to extract the temporal cues for pitch perception. It is a fundamental requirement of STDP that the pre- and post-synaptic spike times be present within a limited time window [32], whose time course is measured by electrophysiology. Evidence shows that the contribution of pre-synaptic/post-synaptic spike pairs to learning vanishes faster than the post-synaptic/pre-synaptic spike pairs [33]. In other words, depression lasts longer than potentiation. In this study, this condition was satisfied by applying an asymmetric learning window with a wider extent towards depression,

$$W\left(s\right)=\;\{\begin{array}{c} A_{p}\;\text{exp}\;\left(\frac{s}{\tau_{p}}\right)\;,\;s>0\\ -A_{d}\;\text{exp}\;\left(\frac{s}{\tau_{d}}\right)\;,\;s<0, \end{array}$$ (5)

where *s* is the post-synaptic spike time minus the pre-synaptic spike time and *τ*_*p*_ and *τ*_*d*_ are the time-constants for potentiation and depression, respectively. *A*_*p*_ and *A*_*d*_ are constant gains for potentiation and depression, respectively. The shape of this particular learning window is shown in Fig 1G. Chosen parameters for the learning window in this paper are *A*_*p*_ = 15 and *τ*_*p*_ = 1 ms, as gain and time-constant, respectively, for potentiation (*s* > 0) and *A*_*d*_ = 10 and *τ*_*d*_ = 5 ms, as gain and time-constant, respectively, for depression (*s* < 0).

Although the time-constants of the learning window used in this study were much shorter than those reported for hippocampal neurons by Bi and Poo [27] (viz., *τ*_*p*_ = 17 ms and *τ*_*d*_ = 34 ms), studies have shown that the auditory pathway has specialized neurons that enable the processing and transmitting of fine-grained temporal information. For example, Gerstner et al. [30] used time-constants as short as 0.5 ms in their model of the barn owl sound source localization system.

The STDP rule considered in this study modified the synaptic efficacies based on two terms according to

$$\Delta w_{ij}=\;\eta \left(\int_{0}^{T}\int_{-t}^{T-t}W\left(s\right)\;S_{i}\left(t\right)S_{j}\left(t+s\right)ds\;dt-b_{j}\int_{0}^{T}S_{j}\left(t\right)dt\right),$$ (6)

where *S*_*j*_ and *S*_*i*_ are the spikes of the pre- and post-synaptic neurons, respectively, *W*(*s*) is the learning window, *T* is the learning time, and *η* is the learning rate. *b*_*j*_ is a constant coefficient specifying the contribution of the pre-synaptic neurons to changing the weights. The first term on the right-hand side of (6) corresponds to the temporal correlations between the pre- and post-synaptic neurons and is responsible for shaping the neural structure (by selecting correlated synapses), while, with an appropriate *b*_*j*_, the second term on the right-hand side of (6) maintains the post-synaptic spiking rate within a defined regime.

The learning proceeds for a much longer time than the temporal extent of the learning window so the learning window integral limits can be changed to infinity with a minor error and the spike-counting integrals in (6) can be replaced by temporal averages, i.e., $\langle \bar{S_{i}\left(t\right)S_{j}\left(t+s\right)}\rangle$ and $\langle \bar{S_{j}\left(t\right)}\rangle$ [34]. When the temporal correlation between the pre- and post-synaptic neurons is insignificant, the ensemble temporal average, $\langle \bar{S_{i}\left(t\right)S_{j}\left(t+s\right)}\rangle$, can be estimated by individual temporal averages, $\langle \bar{S_{i}\left(t\right)}\rangle \langle \bar{S_{i}\left(t+s\right)}\rangle$. Then (6) can be simplified to a rate-based learning rule given by

$$\Delta w_{ij}=\;\alpha \left(\nu_{i}-\;\bar{\nu }\right)\nu_{j},$$ (7)

where *ν*_*j*_ and *ν*_*i*_ are the pre- and post-synaptic spiking rates, respectively, $\bar{\nu }$ is the desired average post-synaptic spiking rate, and *α* is the learning rate. For α < 0, (7) modifies the synaptic weights in order to maintain *ν*_*i*_ close to $\bar{\nu }$ [34]. Similarly, for a negative learning window integral (we set $\int_{-\infty }^{+\infty }W\left(s\right)ds=A_{p}\tau_{p}-A_{d}\tau_{d}=-0.035$) and $b_{j}=\bar{\nu }\int_{-\infty }^{+\infty }W\left(s\right)ds=-1.05$, (6) keeps the post-synaptic spiking rates at around $\bar{\nu }=30$ spike/s. Furthermore, applying the learning window would result in strengthening correlated synapses, which is shown in the Neural learning section of the Results to lead to structure formation corresponding to pitch.

The synaptic connections for each pitch neuron, *i*, were modified by STDP in the presence of a sound with the same pitch as the pitch neuron’s dedicated label. For example, sounds with 110 Hz pitch were presented to the Poisson neurons and the connections of the 110 Hz pitch neuron were subsequently adjusted by STDP. Learning time for each pitch category was 5000 s (= learning time, *T*). The initial synaptic weights for all the input/output connections were set to a fixed value, *w*_0_ = 0.0075. This resulted in high spiking rates (~80 spike/s) in LIF neurons for all the pitch categories. The advantage of inducing a high initial spiking rate was that it led to faster plasticity due to more spikes falling within the learning window. Synaptic weights were restricted to remain in the [*w*_*min*_, *w*_*max*_] range.

For a better exploration of the model’s dynamics in response to different spectral shapes, single-type and mixed-type STDP learning were implemented. In the former, all the pitch categories were learned exclusively through a single type of stimuli. Single-type learning was simulated for pure tone, vowel, and piano stimuli because these types had samples for all the pitch categories. In the mixed-type stimuli, for each pitch category, a stimulus type (pure tone, vowel, and the four musical instruments) was chosen randomly and the corresponding spatio-temporal pattern was used as training material. Ideally, a full mixed-type learning would require each pitch category to be learnt through all the existing stimulus types, however, this might not be the case in a real world listening environment. To make the learning process more realistic and at the same time provide sufficient spectral variations to the model, mixed-type learning was repeated five times–with different randomly chosen types—and the corresponding emerged dynamics were averaged.

For a complete list of Phase II parameters see Table 2. The LIF neurons and STDP parameters (except for the learning window potentiation and depression time-constants) were taken from previous studies by Kerr et al. [35,36]. The spiking neural network was simulated and trained with STDP using an in-house C++ program called “SpikeSim” [35,37].

**Table 2:** Spiking neural network and learning parameters.

**Type**	**Parameter**	**Notation**	**Value**
Neuron	Membrane time-constant	*τ*_*m*_	10 ms
	Threshold potential	*V*_*th*_	-50 mV
	Resting potential	*V*_*p*_	-65 mV
	Reset potential	*V*_*r*_	-65 mV
	Reversal potential	*V*_*rev*_	0 mV
	Refractory period	*t*_*ref*_	1 ms
Learning	Initial synaptic weights	*w*_0_	0.0075
	Upper-bound for weights	*w*_*max*_	0.2
	Lower-bound for weights	*w*_*min*_	-0.2
	Synaptic delay	Δ	10 ms
	EPSC rise time	*τ*_*A*_	0.5 ms
	EPSC decay time	*τ*_*B*_	1 ms
	Potentiation time-constant	*τ*_*p*_	1 ms
	Depression time-constant	*τ*_*d*_	5 ms
	Window height (potentiation)	*A*_*p*_	15
	Window height (depression)	*A*_*d*_	10
	Contribution of the input spikes	*b*_*j*_	-1.05
	Learning rate	*η*	10^−7^
	Learning time	*T*	5000 s
	Desired post-synaptic rate	$\bar{\nu }$	30 spike/s

## Results

### Neural learning

**Fig 3:**
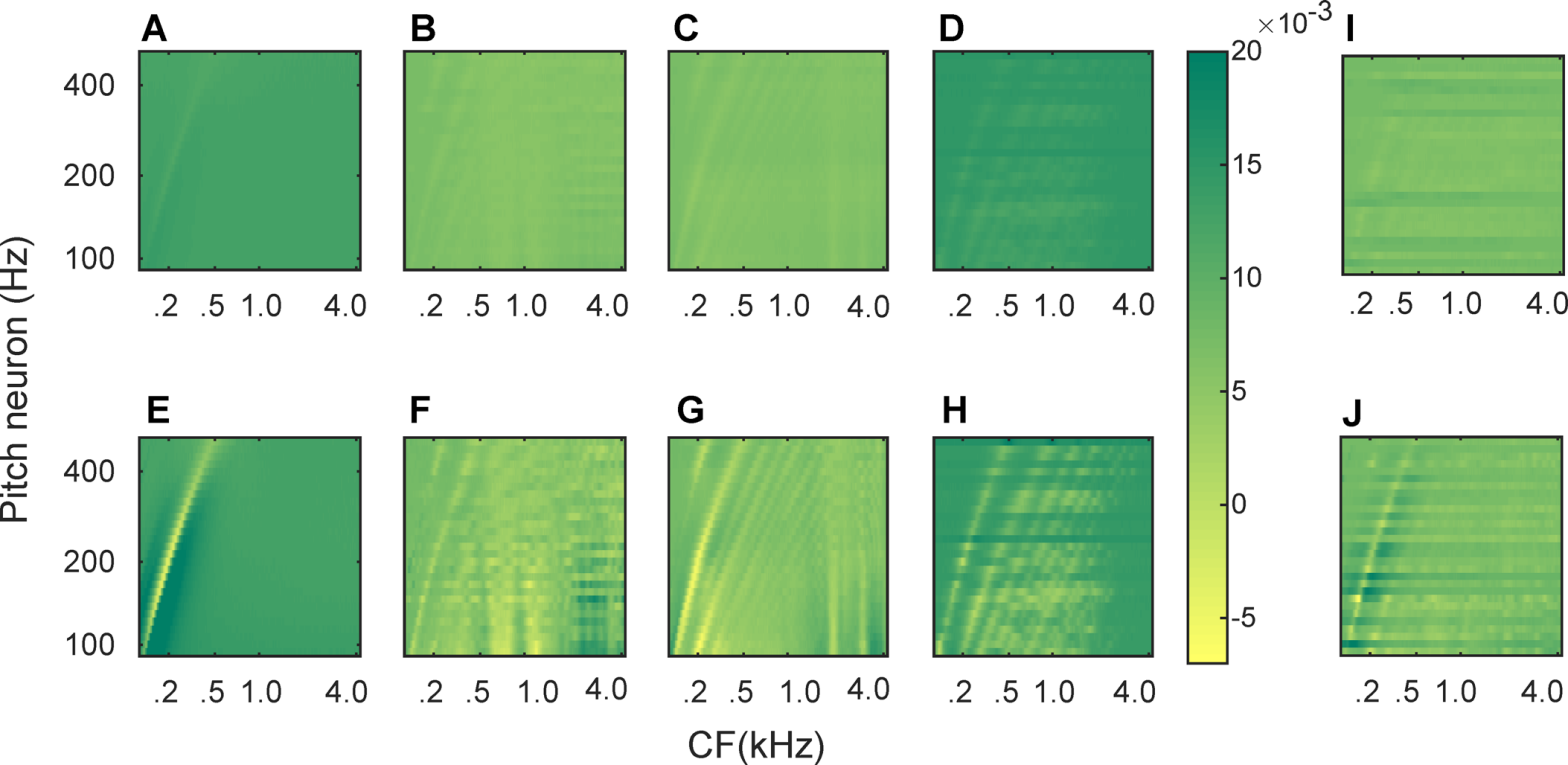
Plots of synaptic weight patterns at an initial and a final stage of STDP learning. (A) Patterns recorded after 500 s of learning with pure tone stimuli. (B) Patterns recorded after 500 s of learning with /ɑ/ stimuli. (C) Patterns recorded after 500 s of learning with /i/ stimuli. (D) Patterns recorded after 500 s of learning with piano keys. (E) Patterns recorded after 5000 s of learning with pure tone stimuli. (F) Patterns recorded after 5000 s of learning with /ɑ/ stimuli. (G) Patterns recorded after 5000 s of learning with /i/ stimuli. (H) Patterns recorded after 5000 s of learning with piano keys. (I) Patterns recorded after 500 s of learning with mixed stimuli. (J) Patterns recorded after 5000 s of learning with mixed stimuli. In (A-J) CFs of input neurons are presented along the abscissa and the ordinate shows the pitch category that each output neuron represents. The colormap is consistent among all graphs.

During the course of learning, synaptic weight changes directed by STDP gradually decreased the initial spiking rate for each pitch neuron to an asymptote rate of ~30 spike/s. Synaptic weights were recorded as the learning progressed. Fig 3 shows the input/output connectivity patterns (*w*_*ij*_) that developed for different types of stimuli at an initial (top row) and a final (bottom row) stage of learning. Graphs (A-D) are associated with the weight patterns recorded after 500 s presentation of pure tones, /ɑ/ vowels, /i/ vowels, and piano sounds, respectively, examples of each were shown in Fig 2A–2D. In each graph, input neurons (*j* index) are shown along the abscissa, sorted by their CF (in kHz). Output or pitch neurons (*i* index) are presented along the ordinate, sorted based on the pitch that they represent. Graphs (E-H) show the corresponding emerged patterns when learning progressed for 5000 s. Fig 3I and 3J show the average synaptic weight patterns that emerged after 500 s of mixed-stimulus learning and after 5000 s of mixed-stimulus learning, respectively.

Because pitch categories, as opposed to spectral shapes, were consistent during the course of learning leading to the patterns in Fig 3E–3H, it could be inferred that the common behavior observed amongst the four patterns in Fig 3E–3H would be associated with pitch. Therefore, the “wrinkle” (peak-trough-peak sequence) that started from the bottom-left and moved towards the top-middle in each graph would correspond to pitch. In all the four patterns, for each pitch neuron, the trough of the wrinkle appeared at input neurons with CFs similar to the pitch categories. This “pitch curve” was the only apparent feature in pure tone patterns (Fig 3E) due to their simple spectra. Vowels (Fig 3F and 3G), on the other hand, had spectral power concentrated around the formants. Connections originating from formant locations were strongly affected by STDP due to high driving rates. Formants thus resulted in vertical stripes in the synaptic patterns in Fig 3F and 3G. As shown in Fig 2L, piano stimuli led to a distinct harmonic structure with evenly-distributed energy across the low-frequency half of the cochlear regions, which resulted in harmonically-related pitch patterns (Fig 3H). The timbre-independent pitch curve was replicated by the mixed-stimuli model (Fig 3J) as well; however, due to various spectral shapes presented to the model during learning, type-specific behavior observed in Fig 3F–3H is absent in the weight patterns of the mixed-stimuli model.

### Temporal adjustments

**Fig 4:**
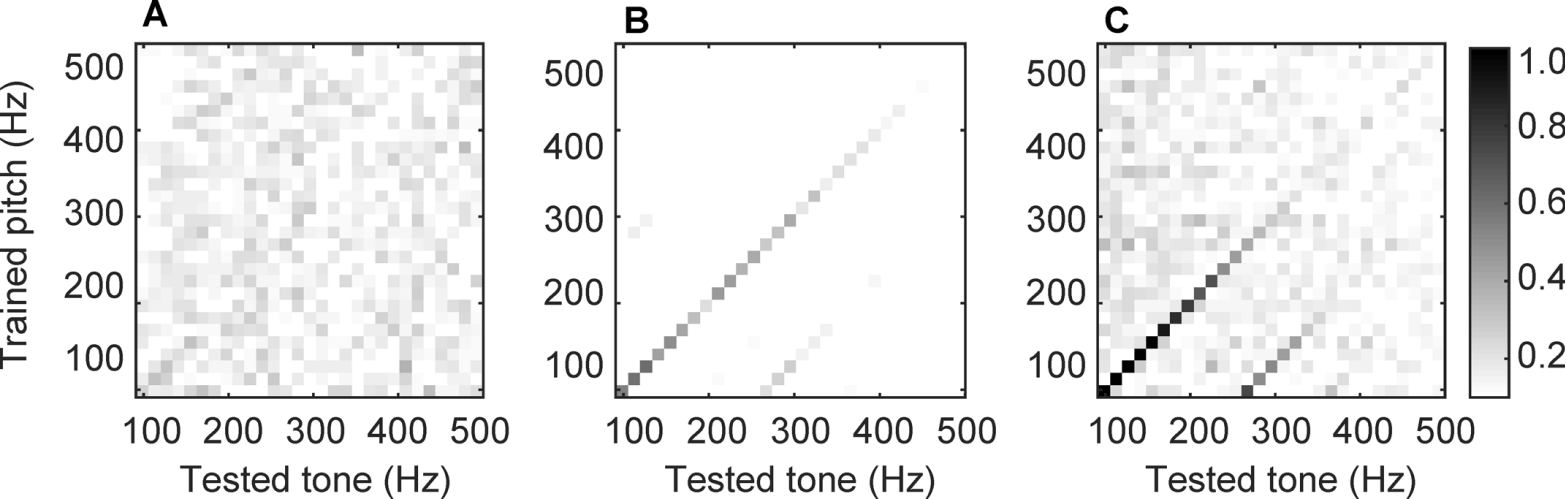
The effect of learning on pitch neurons’ spike timings. (A) Matrix of vector strength for an initial 5 s intervals during mixed-stimuli learning. (B) Matrix of vector strength for a final 5 s interval during mixed-stimuli learning. (C) Matrix of vector strength for a final 5 s interval during learning with high-pass filtered vowels only.

In order to measure the efficiency of STDP in adjusting the spike timings (e.g., in terms of producing phase-locked responses), vector strength was calculated for pitch neurons during early and late stages of learning. Vector strength is a well-known measure of phase locking or stimulus-response synchrony; it describes a phase relationship between the periodic input stimuli and the discharge of the output neuron [38]. Fig 4 presents vector strength matrices (stacked vector strengths from all the pitch neurons) computed from 5 s initial (A) and final (B) intervals, using the mixed-stimuli model. According to the noisy pattern of Fig 4A, during the early stages of learning − when the initial uniform synaptic weights had not been modified by the plasticity rule–the pitch neurons generated spikes at random times. However, after sufficient learning (Fig 4B), it was observed that for each pitch neuron, vector strength was strongest for the input stimuli that had the same pitch as that represented by the pitch neuron (the diagonal lines). This indicated that STDP had adjusted the connection strengths so that the spikes were more likely generated in-phase with a sinusoid of the same frequency, i.e., one spike per sinusoidal peak.

A question was then posed as to whether the temporal adjustment of the spikes would be affected by the absence of F0. To investigate this matter, in another simulation, the spiking neural network was exclusively presented with high-pass filtered vowels during the course of STDP learning. Fig 4C shows the resulting vector strength matrix for a final 5 s interval. It was observed that spike times became entrained to F0 by STDP, even when F0 was missing.

### Extracting the temporal cues

The inter-spike-interval histogram (ISIH), has proven to be an efficient measure of pitch, compatible with pitch-related psychophysical findings for a wide range of stimuli and levels [23]. Cariani and Delgutte [23] found that peak locations and relative amplitudes in a histogram of inter-spike-intervals provided a cue for pitch that was robust against sound level changes and spectral shape variations. The latter thus provided an explanation for pitch constancy at a neural level.

In this study, the most frequent or the dominant interval was considered as the temporal code of pitch. Although deriving the most common interval was possible by taking into account the spiking activity of a single pitch neuron [39], it was decided to use the ISIH of the population of pitch neurons (a.k.a., pooled ISIH) to account for the role of higher-order pitch processing centers in integrating information across pitch neurons. This was necessary to explain phenomena such as perception of the missing-F0 pitch that reportedly engages higher-order auditory processing centers [40].

**Fig 5:**
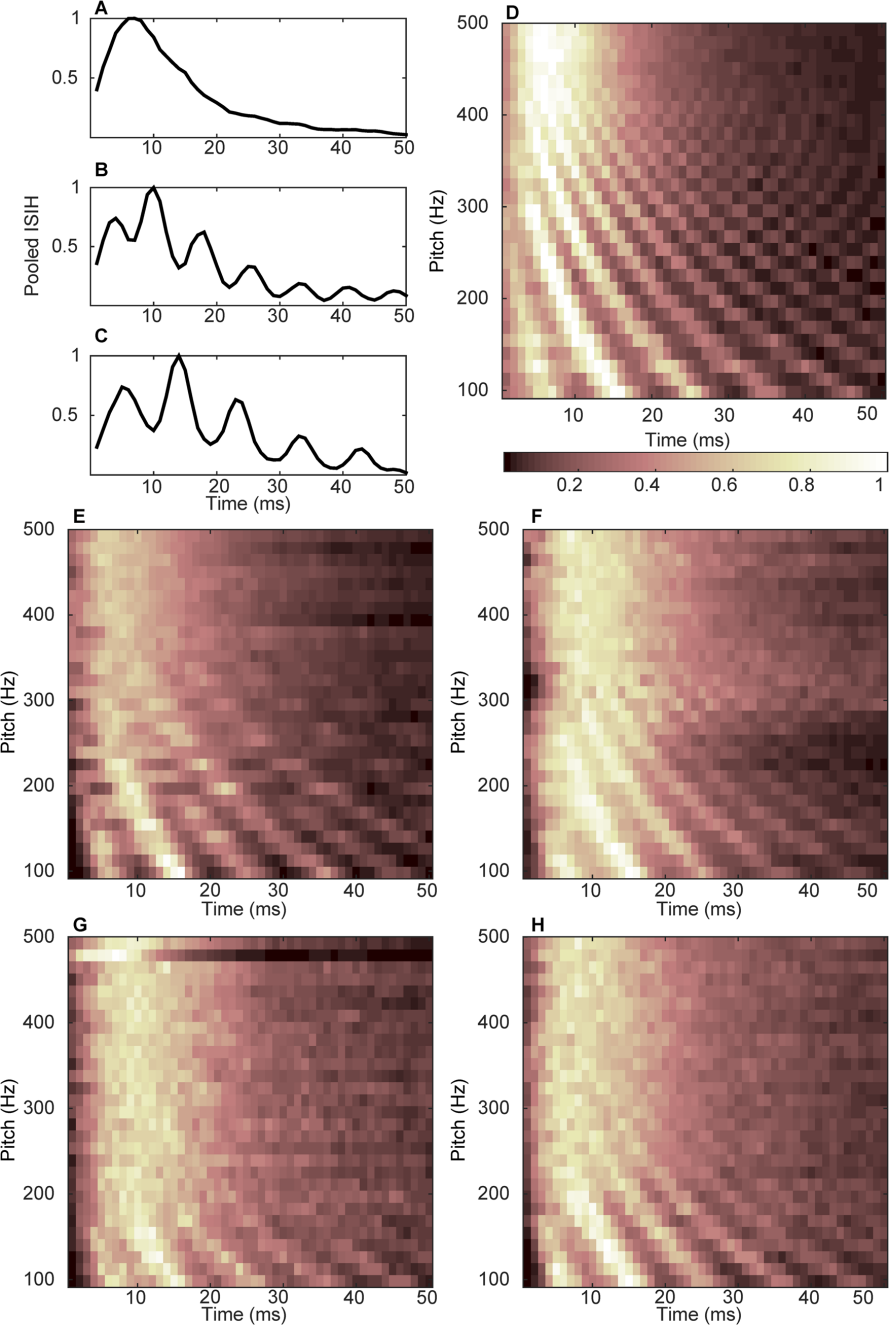
Pooled ISIH for different types of stimuli. (A) Histogram associated with the pitch category of 370 Hz (pure tone). (B) Histogram associated with the pitch category of 131 Hz (pure tone). (C) Histogram associated with the pitch category of 104 Hz (pure tone). (D) Stacked pooled ISIH for the 29 pitch categories of pure tones. (E) Stacked pooled ISIH for the 29 pitch categories of /ɑ/ vowels. (F) Stacked pooled ISIH for the 29 pitch categories of /i/ vowels. (G) Stacked pooled ISIH for the 29 pitch categories of high-pass filtered /ɑ/ vowels. (H) Stacked pooled ISIH for the 29 pitch categories of high-pass filtered /i/ vowels. Pitch categories are shown on the ordinate and ISIH amplitudes are shown in color in D-H. Histograms are slightly smoothed and normalized to maximum for better visualization in (A-H).

To calculate the ISIH for each pitch category, the mixed-stimuli trained model shown in Fig 3J was presented with each of the 29 pure tones for 0.5 s. The resulting inter-spike intervals were pooled across the 29 pitch neurons and distributed in 1 ms bins. Fig 5A–5C shows examples of the first 50 ms of the pooled ISIHs for pitch categories of 370 Hz, 131 Hz, and 104 Hz, respectively. For better visualization, all the histograms were smoothed (using a moving average filter with a span of three) and normalized to maximum. Pitch values presented in Fig 5A–5C were selected as representatives of high-, medium-, and low-pitch stimuli in order to demonstrate how the temporal pitch information changed as a result of pitch increase.

A stacked pooled ISIH graph was generated by accumulating all the 29 pooled ISIHs (e.g., Fig 5A–5C), arranged by the stimulus pitch. The stacked pooled ISIH is shown in Fig 5D, with pitch categories shown along the ordinate in Hz and histogram amplitude represented by color. Similar stacked pooled ISIH are shown for vowels /ɑ/ and /i/ in Fig 5E and 5F, respectively. Fig 5G and 5H show the stacked pooled ISIHs for high-pass filtered vowels /ɑ/ and /i/, respectively.

It was observed that for all stimuli types, as the pitch of stimuli increased, the amplitude and the number of histogram peaks became stronger and fewer, respectively, indicating that the model used shorter inter-spike-intervals (viz., rapidly-occurring spikes) to encode higher pitches. Stacked histograms thus provide a representation of how the model temporally processes the pitch.

### Pitch perception using temporal cues

To demonstrate the effectiveness of the temporal cues in providing pitch information, a pitch ranking model using the pooled ISIHs as input was simulated. Pitch ranking is a typical psychophysical experiment wherein listeners are asked to decide which of the two presented sound stimuli has a higher pitch. Normal-hearing humans score about 70%-100% depending on the pitch difference in a sound pair and type of stimuli. For example, at one-semitone pitch difference using sustained vowels, subjects scored about 81% [41], which increased to about 100% when the pitch difference was increased to six semitones.

**Fig 6:**
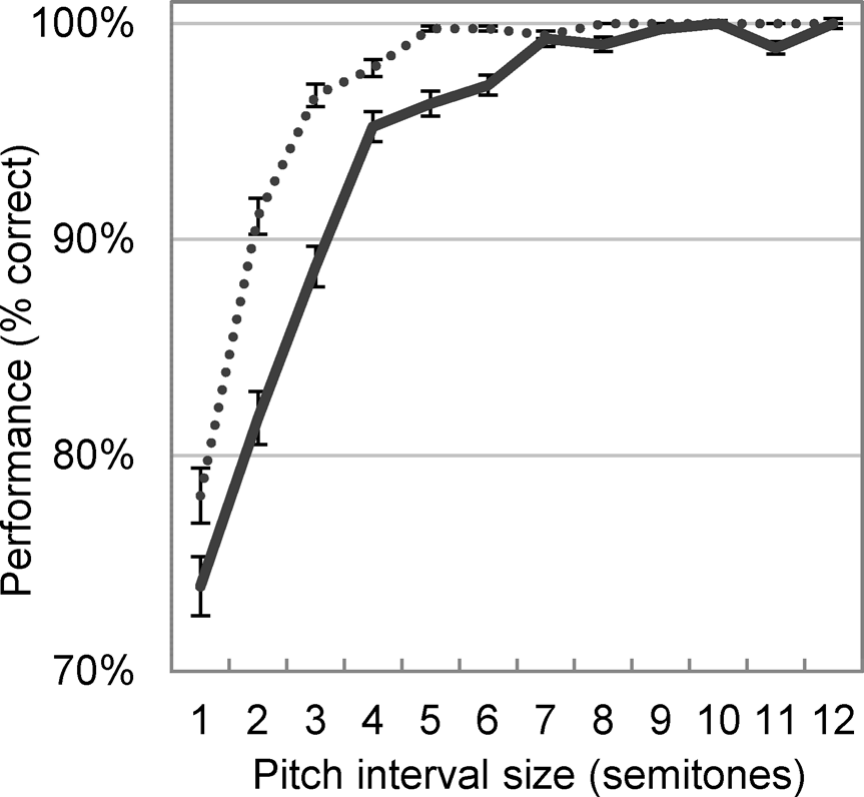
Simulated pitch ranking scores at different pitch interval sizes. Model performance using the extracted ISIHs from original (i.e., Fig 5E and 5F) and high-pass filtered (i.e., Fig 5G and 5H) vowels are shown with solid and dotted lines, respectively. Error bars show standard errors of the means within simulations trials and where not visible, indicate a very small standard error. Chance level is 50%.

The pitch ranking model in this study consisted of an artificial neural network (a single layer perceptron with two neurons) that received two sets of inputs corresponding to a pair of stimuli and generated two outputs, the higher of which would indicate the higher-pitch stimulus. For 20 trials, the model was trained on 1500 pitch pairs (10% reserved for validation) and tested on 500 unseen pitch pairs. Performance at each trial was computed as the number of correct answers divided by the total number of presentations (i.e., 500). At each trial, pitch pairs were selected randomly from a pool of all eligible combinations of vowel stimuli. For a fair comparison between simulated results and available psychophysical data, only same-type vowel pairs (e.g., /ɑ/-/ɑ/ and /i/-/i/) with pitch differences between one and twelve semitones were allowed in the pool. The weights of the artificial neural network were adjusted using the error back-propagation method [42]. The overall performance of the model was calculated as the average performance over the 20 trials. The exact same simulations were performed using the high-pass filtered vowels. Fig 6 presents the overall performance of the model as a function of pitch difference for the original and high-pass filtered vowels.

## Discussion

Humans are born with some pitch perception abilities [43,44]. For example, through measuring event-related potentials, Leppänen et al. [43] reported that newborns were able to detect pitch changes in sequential tones. However, perceiving the missing-F0 pitch does not happen until 3–4 months of age [45]. He and Trainor [45] concluded that unlike pure tones and complete harmonic complexes that possibly relied only on a *peripheral* representation of the stimulus, processing the pitch of missing-F0 stimuli required *cortical* engagement to integrate the information from across the auditory periphery and elicit a single pitch percept. Auditory cortical development is an unsupervised process that happens naturally during early infancy.

In this study, it was observed that a correlation-based, unsupervised, spike-based form of Hebbian learning could explain the development of the neural structure required for recognizing the pitch of simple and complex tones, with or without F0. The emerged neural structure led to precisely-timed responses that were necessary for a reliable population code for pitch. More specifically, the synaptic wrinkles (Fig 3J) constituted a mechanism to compensate for the travelling wave delay that was the main cause of temporal misalignment between the spikes coming from different cochlear positions. Similar compensatory mechanisms (i.e., through developing proportional dendritic delays) were found by Greenwood and Maruyama [7] and Oertel et al. [8] in the cochlear nucleus.

Another interesting finding of this study was that although the emerged synaptic connection patterns followed the spectral power of the signal (i.e., the rate profiles), which varied amongst different stimulus types (Fig 3E–3H), the ISIH pattern extracted from the mixed-stimuli learning (Fig 5D–5H) emerged regardless. In other words, the temporal pattern shown in Fig 5D–5H would appear for any type of sound source, given that the model has experienced sufficient variations of the spectral shapes. It can thus be concluded that the temporal code for pitch could successfully extract invariances (F0) among inputs, although the inputs were spectrally different. The temporal code of pitch, therefore, can explain the pitch constancy phenomenon.

From a computational standpoint, the resemblance between the rate profiles (Fig 2I–2L) and the evolved synaptic weight patterns (Fig 3E–3H) indicated that STDP was mainly driven by the average activity or spiking rate of the pre-synaptic (auditory) neurons. However, applying the learning window enabled this correlation-based learning rule to incorporate temporal precision and generate responses that were indicative of pitch, regardless of the spectral shape of the stimulus. That is, the learning algorithm could successfully compensate for rate-place inconsistencies among different types of stimuli and provide a rate-independent temporal code. The ability of the model to replicate the above-mentioned phenomenon was of special importance because finding a spectrum-independent code for pitch has been considered a substantial step forward in the research field of pitch perception [46]. Neural correlates for “pitch constancy” have been detected in the auditory cortex of primates by Bendor and Wang [1]. They found that the pitch selective neurons would respond to both pure tones and harmonic complexes of the same pitch, even when F0 was eliminated from the latter’s spectrum. Simulated cortical columns in this study, therefore, could be considered as a computational substrate for what Bendor and Wang [1] labelled as pitch neurons.

As demonstrated in Fig 2J and 2K, eliminating the low-frequency content of the vowels led to flat lines in the place code corresponding to low-CF neurons. As Fig 4C suggested, STDP was still able to fine-tune the timing of the spikes, despite the missing fundamental. Compared to the original vowels, high-pass filtering the vowels did, however, lead to a noisier vector strength matrix (Fig 4B vs. 4C), indicating that entraining the spikes to a correct phase became a more challenging task for STDP when the fundamental frequency was not available in the spectrum. This was nevertheless an issue when the spiking neural network learnt mixed-stimuli due to exposure to many more representations and spectral variations. The absence of the fundamental frequency did not impair the performance of the pitch ranking model (Fig 6), confirming that the extracted temporal cues were independent of the fundamental frequency.

### Advancing the modelling field

The place and the temporal codes of pitch are the roots of current pitch perception models, dividing the modern models into pattern matching- and autocorrelation-themed classes [47], respectively. The former estimates pitch based on a pattern or template, which is normally derived from an auditory model simulating the frequency analysis of the cochlea. Better-known examples of this category are the harmonic sieves model of Cohen [48] and the harmonic templates of Shamma and Klein [5]. The autocorrelation class, however, requires self-similarity measures, such as autocorrelation, to estimate periodicity. Examples of this category include Licklider’s [49] duplex theory-themed models such as the ones developed by Meddis et al. [50] and Patterson et al. [3].

The pitch perception model developed in this study employed elements of both modelling approaches in a more biologically-plausible platform. In fact, the present model followed closely the schematic pitch perception model suggested by Moore [51] that also combined the place and temporal code of pitch to explain how the pitch of complex sounds might be perceived by the auditory system. Similar to the model presented in this study, Moore’s schematic model also consisted of a bank of cochlear filters (similar to Fig 1E), a spike generation process (Poisson neurons in Fig 1F) and a spike analyzer that computed the pooled ISIH. A final decision making step would pick the most prominent interval as an estimate of the stimulus period. The learning phase employed in the current model, additionally provided a description of the development of the neural structure leading to the required ISIHs. The learning phase would also provide a biological analogue to Shamma and Kleins’ detector units [5], as well as eliminating the need for long neural lags in autocorrelational models.

It should be noted that the current model could reproduce the inter-spike interval statistics similar to the actual auditory nerve recorded by Cariani and Delgutte [23] and taken into account in Moore’s [51] model. However, the artificial neural network that made pitch judgments based on the received ISIHs was a simple model to demonstrate the effectiveness of the temporal cues in performing a simple pitch perception task and was not intended to be a biologically-plausible model of higher-order auditory system. In addition, in future work, the STDP learning step would present all pitches to all neurons, with a soft winner-take-all mechanism implemented to achieve competition between the neurons to create the pitch map across them.

### Implications for cochlear implant research

The cochlear implant or “Bionic Ear” is one of the most successful neural prosthesis that restores partial hearing in profoundly deaf people by directly stimulating the auditory nerve with controlled electrical current pulses. Many implantees have obtained functional speech perception in favorable conditions similar to their normal hearing peers [52]. However, there are still unresolved issues like tone perception in tonal languages and speech perception in noisy environments [53,54]. Music melody appreciation is also very limited in cochlear implant users [55]. It has been shown that pitch perception in implant hearing is correlated with the users’ abilities in performing the abovementioned tasks [56]. Accordingly, if pitch perception is improved in cochlear implant patients, their auditory performance should also get better.

Similar to normal hearing, pitch information in multi-channel cochlear implant hearing is also carried through place and temporal cues [55,57]. In electrical hearing, place cues for pitch perception are associated with the tonotopically-arranged electrodes. For example, Nelson et al. [58] reported that the pitch elicited by stimulating basal electrodes was generally consistently higher than that of the apically-located electrodes. On the other hand, the rate of stimulation and the frequency of amplitude-modulation of the stimulation pulses have impacts on the perceived pitch that could only be explained by the temporal cues for pitch perception. For example, Tong et al. [59] found that, in a cochlear implant listener, high-rate stimulation (in an isolated electrode) resulted in a high-pitch sensation and vice versa. The modulation frequency has a similar effect on pitch as that of the rate of stimulation [60,61].

Although cochlear implants are able to induce cues for pitch perception similar to those used by normal hearing listeners, the quality of the cues is considerably limited in electrical hearing. For instance, a limited number of electrodes and depth of electrode insertion confine the place cues to a limited frequency range [62,63]. Moreover, the tonotopic order in electrical hearing may be distorted in cochlear implant subjects (e.g., Schatzer et al. [64]), resulting in a poor frequency-to-place mapping. Temporal cues are also restricted to a cap rate of about 300 Hz in cochlear implant hearing. This means that stimulation rates or modulation frequencies above this limit do not induce distinctive pitch percepts [59,65,66].

Due to limited depth of electrode array insertion and implant filters suppressing the low-frequency content of the signal (lowest band-pass filters in cochlear implants have a center frequency of ~125 Hz), cochlear implants are not normally able to convey F0 information through the place code. From this point of view, hearing through a cochlear implant is analogous to hearing through a telephone transmission line. The results of this study showed how normal hearing listeners could perceive the missing-F0 pitch by using the temporal cues. Therefore, it can be inferred that improving the temporal cues in cochlear implant users may compensate for the impaired place cues and eventually lead to a better pitch perception. Application of a pitch perception model using the place code in evaluating the effect of stimulation field spread on pitch perception in cochlear implant hearing can be found in a study by Erfanian Saeedi et al. [67]. Similarly, with a modified auditory periphery (Fig 1E), the model developed in this study can be used to estimate the efficiency of experimental sound processing strategies (e.g., [68,69]) in terms of providing better temporal pitch perception cues. Extending the application of the current model to cochlear implant research would require replacing the normal hearing cochlear filters with descriptors of auditory neuron responses to electrical stimulation, examples of which can be found in [70–74].
